# Neutrophil extracellular traps involved in the pathogenesis of IgA vasculitis: Confirmed in two IgAV rat models

**DOI:** 10.1371/journal.pone.0288538

**Published:** 2023-07-21

**Authors:** Xiu-Qi Chen, Jia-Sen Zou, Li Tu, Xiang Yun, Yuan-Han Qin

**Affiliations:** Department of Pediatrics, The First Affiliated Hospital, Guangxi Medical University, Nanning, China; University of Bari: Universita degli Studi di Bari Aldo Moro, ITALY

## Abstract

**Background:**

Neutrophil extracellular traps (NETs) have been found to play a role in the development of autoimmune diseases. In the past two years, studies have demonstrated a significantly increase of NETs in skin tissues during the early stages of IgAV, indicating their involvement in disease activity among children with IgAV. However, the presence of NETs in IgAV animal models has not yet been reported. The objective of this study is to investigate whether NETs are involved in the pathogenesis of IgA vasculitis (IgAV) rats.

**Methods:**

Twenty-four SD rats were randomly divided into three groups: the ovalbumin group, the gliadin group, and the control group. The IgAV rat models were established administering Indian ink with ovalbumin (ovalbumin group) or gliadin (gliadin group) with Freund’s complete adjuvant. The cell-free DNA (cf-DNA) was quantified by using dsDNA quantification kit, while the levels of Immunoglobulins, complement C3 and myeloperoxidase-DNA (MPO-DNA) in serum were tested using enzyme linked immunosorbent assay (ELISA). The IgA, complement C3 and NETs in tissues were detected through multiple immunofluorescences.

**Results:**

Both the ovalbumin group and gliadin group showed IgA and C3 deposition in various tissues, including the glomerular mesangial region, skin, and digestive tract, while the control group showed no such deposition. The levels of circulatory cf-DNA and MPO-DNA, which are components of NETs, were significantly elevated in both ovalbumin and gliadin groups compared with the control group. Furthermore, the presence of NETs were found in gastrointestinal and renal tissues of the ovalbumin and gliadin groups, but not in the control group.

**Conclusions:**

IgAV model rat can be established through the combination of ovalbumin and gliadin with Indian ink and Freund’s complete adjuvant. This study provides the first confirmation that NETs are involved in the pathogenesis of IgAV rat.

## 1. Background

Immunoglobulin A vasculitis (IgAV), also known as Henoch‐Schönlein purpura, is a systemic small vessel vasculitis mediated by immune complexes (IC) and characterized by non-hemorrhagic skin purpura, abdominal pain, gastrointestinal bleeding, arthralgia, and glomeruli involvement [1, 2]. The pathological manifestation of IgAV is characterized by the deposition of IgA and complement C3 in the walls of small blood vessels [1]. The pathogenesis of IgAV remains elusive, potentially influenced by genetic and environmental factors as well as immune dysregulation [3–5]. In recent years, extensive immunological abnormalities has been found in IgAV, and abnormal glycosylation in the hinge region of IgAl is considered to be the pathological mechanism of IgAV [2]. IgA1-related antibodies, such as anti-IgAl-endothelial cell antibodies, can cause extensive deposition of IgAl in the small blood vessels in target organs like skin, gastrointestinal tract and kidney. This leads to activation of neutrophils and immune cells which release a plethora of inflammatory factors including tumor necrosis factor α (TNF-α), interleukin 1 (IL1), IL6 and IL8. Inflammatory factors can induce endothelial cell damage, increase capillary permeability, and promote neutrophil aggregation, vascular endothelial damage, and microthrombi formation. Additionally, these factors elicit local tissues inflammatory responses that exacerbate disease progression [6–8]. However, the precise mechanism underlying neutrophil-mediated damage in IgAV remains incompletely elucidated.

Brinkmann et al [9] first reported in 2004 that when the body is infected by pathogens, neutrophils can release cell-free DNA (cf-DNA) as the basic extracellular framework, along with citrullinated histones 3 (citH3), myeloperoxidase (MPO), neutrophil elastase (NE), and antimicrobial peptide LL-37 (CAMP, LL37) during pathogen infection to form net-like structures known as neutrophil extracellular traps (NETs). It has been observed that dysregulation of the balance between formation and clearance of NETs is implicated in the pathogenesis of various autoimmune diseases, including systemic lupus erythematosus (SLE) and anti-neutrophil cytoplasmic antibody-associated vasculitis, and rheumatoid arthritis, where excessive accumulation of NETs occurs [10–12]. A 2020 study demonstrated that NETs were significantly increased in skin tissues in the early stages of IC-mediated small vasculitis, such as allergic vasculitis and IgAV [13]. Our previous study also showed that NETs are involved in disease activity with IgAV children [14]. Another study has indicated a positively correlated between MPO-DNA and IgA levels in IgAV patients [15]. There are still few studies that have clarified the elaborated relationship between NETs and IgAV, particularly the mechanism by which NETs induce tissue damage in IgAV. Therefore, further exploration of the mechanism of NETs mediated tissue damage in IgAV is needed using in animal models. However, to date, there have been no reports on the presence of NETs in IgAV animal models. Thus, this preliminary study aimed to investigate whether NETs are involved in the pathogenesis of IgAV rat models established by two methods.

## 2. Materials and methods

### 2.1 Animals and IgAV models

Twenty-four Sprague Dawley rats (1:1 male: female, aged 4-week-old and weighting between 70-90g) were randomly divided into three groups (ovalbumin group, gliadin group and control group) with 8 rats in each group. All the rats were purchased from the Animal Experiment Center of Guangxi Medical University and housed in a specific pathogen-free (SPF) animal facility.

Both ovalbumin and gliadin model groups of rats were administered Indian ink (4 mg/100g BW, Sigma) via tail vein once per week for 3 weeks. Subsequently, the ovalbumin group received an emulsified solution of ovalbumin (10mg/kg, Sigma) [16], while the gliadin group received an emulsified solution of gliadin (Sigma) by intraperitoneal injection once per week for 6 weeks at a concentration of 0.01mmol/L and pH 7.4. The emulsified solution consisted of an equal volume of Freund’s complete adjuvant (Sigma) and ovalbumin (20 mg/ml) in ovalbumin group or gliadin (dissolved in 6mmol/L hydrogen chloride with the final concentration of 0.1%) in gliadin group. After that, the rats in the ovalbumin group were administered ovalbumin physiological saline liquor (10 mg/mL; 0.25 mL) via tail vein injection, while those in the gliadin group received gliadin (0.1% gliadin dissolved in 0.01mmol/L phosphate buffered saline, pH7.4) once per day for 5 consecutive days. The rats in the control group were administered with an equivalent of physiological saline once a week for 3 weeks via tail vein injection, followed by intraperitoneal injection once a week for 6 weeks. Finally, the control group rats received daily administration of physiological saline at the same dosage for 5 consecutive days. The body hair of all rats was shaved to facilitate observation of rash, activity assessment, and monitoring of water and food intake. At the end of 10^th^ week, all rats were anesthetized, and the blood was collected from the abdominal celiac artery. Tissues from the brain, lung, liver, stomach, duodenum, kidney, and skin were harvested and fixed in 10% formalin solution for subsequent morphological and immunohistochemical analysis.

The experiments were approved by the Ethics Committee of the First Affiliated Hospital of Guangxi Medical University and conducted according to the guidelines of animal welfare of the World Organization for Animal Health.

### 2.2 Hematologic and immunoglobulins of IgA, IgE, IgG, C_3_

Blood samples were collected using an ethylene diamine tetraacetic acid (EDTA) tube for hematologic and serum tubes for immunoglobulins testing. The blood routine was examined using an automatic blood cell analyzer (Mindray, BC-2800vet, China) in accordance with the manufacturer’s instructions.

The serum levels of IgA, IgG, IgE and C3 were detected using enzyme linked immunosorbent assay (ELISA). Specifically, the IgA ELISA kit (Epizyme Biomedical Technology Co., Ltd, Shanghai, China), IgG ELISA kit (Epizyme Biomedical Technology Co., Ltd, Shanghai, China), IgE ELISA kit (Sangon Biotech, Shanghai Chia), and C3 ELISA kit (Sangon Biotech, Shanghai Chia) were utilized for this purpose. Briefly, the standard curves were generated using a double ratio dilution gradient. Plasma samples (1:5, 50μL) and 100μL 1×HRP labeled antibodies were added to 96-well microplates and incubated at 37°C for 30–60 min. Subsequently, TMB chromogenic solution (90μL) was added and incubated at 37°C for 20 min in the absence of light. Finally, termination solution (50μL) was added to stop the reaction after slight mixing for 1 minute. The OD value was measured within 10 minutes after adding the termination solution was, with control zero adjusted based on the blank hole. Subsequently, the OD value of each hole was measured in sequence at a wavelength of 450nm using spectrophotometer (Thermo Fisher spectrophotometer 3020). Finally, the contents of IgA, IgG, IgE and C3 were calculated according to the established standard curve.

### 2.3 Hematoxylin and eosin (H&E) and periodic acid-schiff staining

Tissue samples for histology and immunofluorescence were obtained from formalin-fixed, paraffin-embedded blocks., Hematoxylin and eosin (H&E) staining as well as periodic acid-schiff stains were on 5–7 μm thick sections for general morphology and immuno-histochemical analysis. For hematoxylin and eosin (H&E) staining, tissue sections were deparaffinized in xylene for 20 min and soaked in absolute ethanol for 5 min to remove the xylene. The sections were then stained with hematoxylin for 5 min and eosin for 10 min. Finally, the sections were hydrated by soaking them in 85%, 95% ethanol. For PAS staining, tissue sections were oxidated with periodate acid for 15 min, then stained with Schiff’s reagent for 30 min and hematoxylin for 3 min. The histological changes were observed under light microscope (BX53, OLYMPUS).

### 2.4 Quantification of circulating NETs (cf-DNA and MPO-DNA) level

The cf-DNA was quantified using ds-DNA Quant-iT PicoGreen quantification kit (Invitrogen). Briefly, a standard curve was constructed using a range of dilutions from 1 to 1000 ng/ml of cf-DNA. Plasma samples were diluted to 1:5 and the fluorescence signals were measured using a microplate fluorescence reader (Thermo Fisher Spectrophotometer 1510) with filter settings set at wavelengths of 480 and 520nm for excitation and emission, respectively.

The MPO-DNA was quantified using MPO-DNA Elisa Kit (Cusabio) following the manufacturer’s instructions. Briefly, after preparing the standards and diluted samples (1:5), plates were sealed with an adhesive strip and incubated for 60 min at 37°C. Horseradish peroxidase-conjugated antibody specific for MPO-DNA (100 μl) was added and incubated for another 60 min at 37°C. Then, 90 μl of 3,3’,5,5’-tetramethylbenzidene substrate was added and incubated for a further 20 min at 37°C in darkness. The O.D. absorbance at 540 nm was read in a microplate reader (Thermo Fisher spectrophotometer 3020).

### 2.5 Multiple immunofluorescences staining of IgA, C_3_ and NETs (MPO, NE and citH3)

Multiple immunofluorescences staining were performed in according to the instruction manual for multiple fluorescent immunohistochemical staining kit (Absin, Shanghai, China). The paraffin sections were deparaffinized in xylene for 15 min and subsequently dehydrated using gradient ethanol solutions (85% and 75% respectively) for 5 min each. The sections underwent incubation at a temperature of 60–70°C in EDTA antigen retrieval buffer (pH 8.0) for a duration of 15 min, followed by three washes with PBS (pH 7.4). To block endogenous peroxidase, slides were immersed in a solution containing 3% H_2_O_2_ and incubated at room temperature for a period of 15 min before being washed thrice with PBS. After incubating objective tissues with 3% BSA at room temperature for 30 min, the first primary antibodies (Anti-IgA antibody, Absin, Shanghai, China and anti-C3 polyclonal antibody, Absin, Shanghai, China) were added and left to incubate overnight at 4°C. The samples were treated with secondary antibodies conjugated to horseradish peroxidase (HRP) at room temperature for 50 min in dark. Subsequently, the sections were subjected to incubation with fluorescent amplification signal fluorochromes [CY3-TSA (Servicebio, Wuhan, China), FITC-TSA (Servicebio)] in PBS at 4°C for 50 minutes. Finally, DAPI solution was applied to all sections and incubated at room temperature for 10 min.

Multiple immunofluorescences staining of NETs were performed. and fluorescent images of tissue slices were acquired and analysis according to our previous study [14]. Paraffin sections were subjected to antigen-retrieval by incubation in buffer at 60–70°C for 15 min following dehydration in graded ethanol solutions and rinsed with pH 7.4 PBS. Endogenous peroxidase was blocked using a hydrogen peroxide solution (3%) and the sections were then incubated in darkness at room temperature for 25 min. Samples were blocked with a solution of 2% bovine serum albumin and normal goat serum for 30 min at room temperature, followed by incubation with primary antibodies [NE (1:3000; Abcam, Cambridge, UK), MPO (1:1000; Abcam, Cambridge, UK), and citH3 (1:100; Abcam, Cambridge, UK)] in PBS overnight at 4°C. The samples were then incubated with corresponding fluorescently labeled secondary antibodies CY3-TSA (1:2000, Servicebio, Technology Co. Ltd., Wuhan, China), Cy5 affinipure goat anti-rabbit IgG (1:400, Servicebio), and FITC-TSA (1:1000, Servicebio) in PBS for 50 min at 4°C. Coverslips were mounted on glass slides using prolonged gold anti-fade reagent containing DAPI (Servicebio) for 10 min to counterstain the DNA. The slides were subsequently washed three times with PBS for 5 min each time and then covered with a water-soluble fluorescence mounting medium (Servicebio). Images were acquired using 3 DHISTECH Digital slice scanning system (Pannoramic, 3DHISTECH Ltd., Hungary). NETs as determined by cit-H3 that co-localized with MPO/NE within the tissue.

### 2.6 Statistical analysis

The Shapiro-Wilk test was used to determine the normality of the data, which were expressed as the $\bar{X}$ ± SD. Categorical data were expressed as percentages. Statistical analysis between three groups were performed using one-way ANOVA followed by a post-hoc LSD test for quantitative data. The chi-square test was performed in categorical variables. SPSS 19.0 for Windows (SPSS Inc., Chicago, USA) was used for statistical analysis. A P value of <0.05w as considered as statistically significant.

## 3. Results

### 3.1. General conditions, hematologic parameters, serum immunoglobulins, and complement C_3_ levels in IgAV rat models

**Fig 1:**
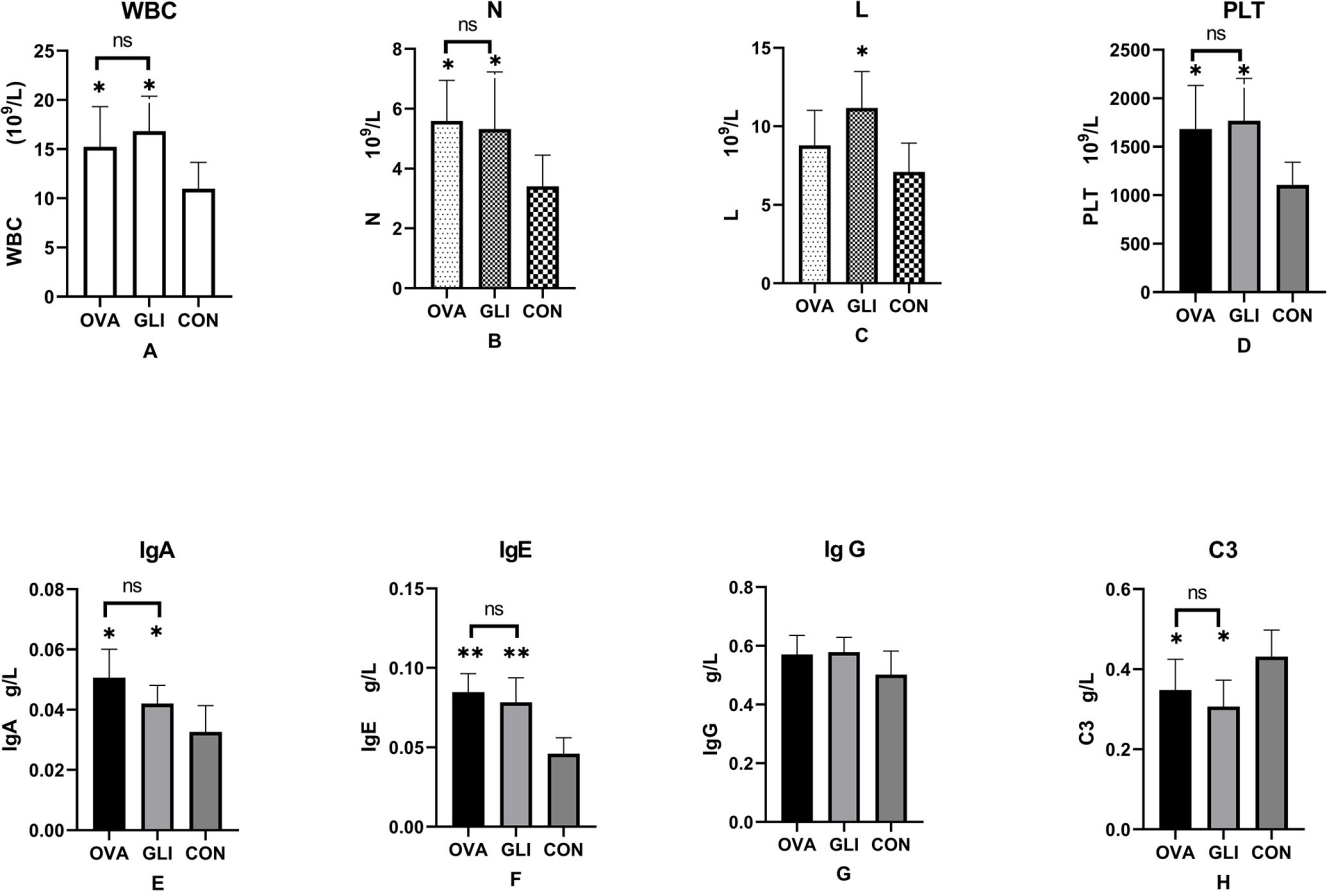
Changes of the hematologic indexes and immunoglobulins in IgAV rat models and control group. ** P<0.01, * P< 0.05. OVA, Ovalbumin group; GLI, Gliadin group; CON, Control group. WBC, White blood cell count. L, Lymphocyte count. N, Leutrophil count. PLT, Platelet count.

For food and water intake, we monitored the dail total consumption. As for activity assessment, we solely examined the differences in standing times among each group. Both ovalbumin and gliadin model groups exhibited varying degrees of reduced activity and intake compared to the control group. In ovalbumin group, 3 out of 8 rats exhibited bleeding spots, while only 2 out of 8 rats exhibited in the gliadin group showed scattered needle-tip sized bleeding spots without any surrounding swelling.The proportion of bleeding spots ranged from approximately 25 to37.5%. In the gliadin group, there was a significant increase in white blood cell (WBC), neutrophil, lymphocyte and platelet compared to the control group (*P* < 0.01). Similarly, in the ovalbumin group, there was a significant increase in WBCs, neutrophil and platelet compared to the control group (*P* < 0.01). However, no significant changes were observed in WBCs, neutrophil, lymphocyte and platelet between both ovalbumin and gliadin model groups (*P* > 0.05) (Fig 1A–1D).

In both ovalbumin and gliadin model groups, there was a significant increase in serum levels of IgA and IgE (*P* < 0.05), while C_3_ levels were significantly decreased compared to control group (*P* < 0.05). However, no significant changes were observed in IgG levels among the three groups (*P* > 0.05). Furthermore, there were no significant differences in all immunoglobulins (IgA, IgG and IgE) as well as C_3_between the ovalbumin and gliadin groups (*P* > 0.05), (Fig 1E–1H).

### 3.2. Immunofluorescence of IgA and C_3_ and pathological changes of IgAV models

**Fig 2:**
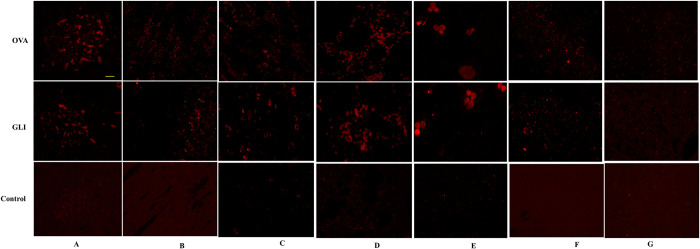
Immunofluorescence of IgA. IgA was positive in the mesangial region of the glomerulus (A), stomach (B), intestine (C), lung (D), skin (E) and brain (F) in both IgAV model groups. The control group was negative. IgA in liver was negative in all the groups. OVA, Ovalbumin group; GLI, Gliadin group. Scale bars 50μm.

**Fig 3:**
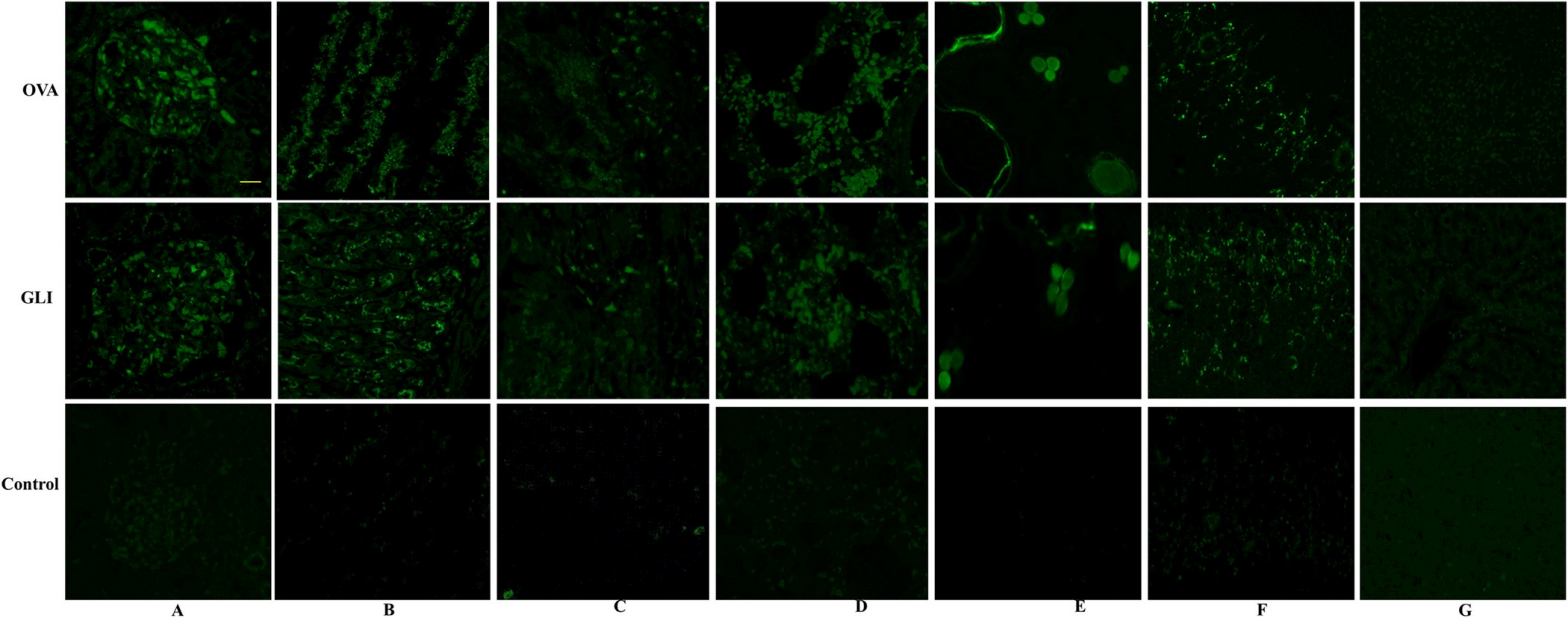
Immunofluorescence of C3. C3 was positive in the mesangial region of the glomerulus (A), stomach (B), intestine (C), lung (D), skin € and brain (F) in both IgAV model groups. The control group was negative. C3 in liver was negative in all the groups. OVA, Ovalbumin group; GLI, Gliadin group. Scale bars 50μm.

**Fig 4:**
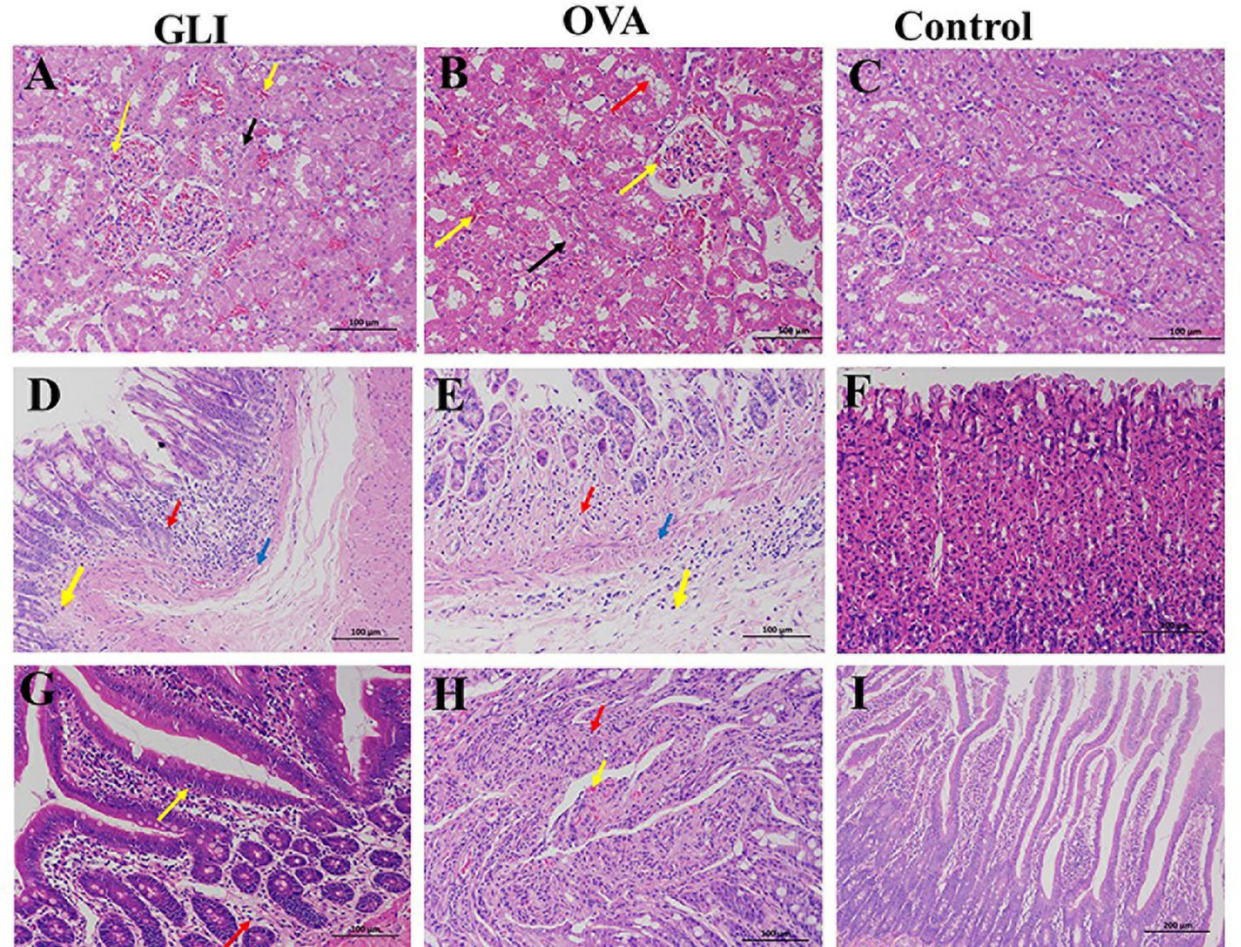
Pathological changes in kidneys, stomach, and intestine of IgAV and control group. OVA, Ovalbumin group; GLI, Gliadin group. Renal tissue: A, B and C. Gastric tissue: D, E, and F. Intestinal tissue: G, H, and I. Numerous renal tubular epithelial cells were denatured, with loose cytoplasm (black arrow), eosinophilic flocculent in the lumen (red arrow). The interstitial and glomerular capillaries were congestion (yellow arrows). The gastric mucosa was bleeding, epithelial cells necrosis (blue arrow), and eosinophil infiltration at the bottom of lamina proper (orange arrow), loose connective tissue arrangement (yellow arrow). The villi of the small intestine were dilated and hyperemic (yellow arrow), and the epithelial cells of the mucous membrane was exfoliated and exposed the lamina propria (black arrow). All the tissues were accompanied by inflammatory cell (red arrow) infiltration.

**Fig 5:**
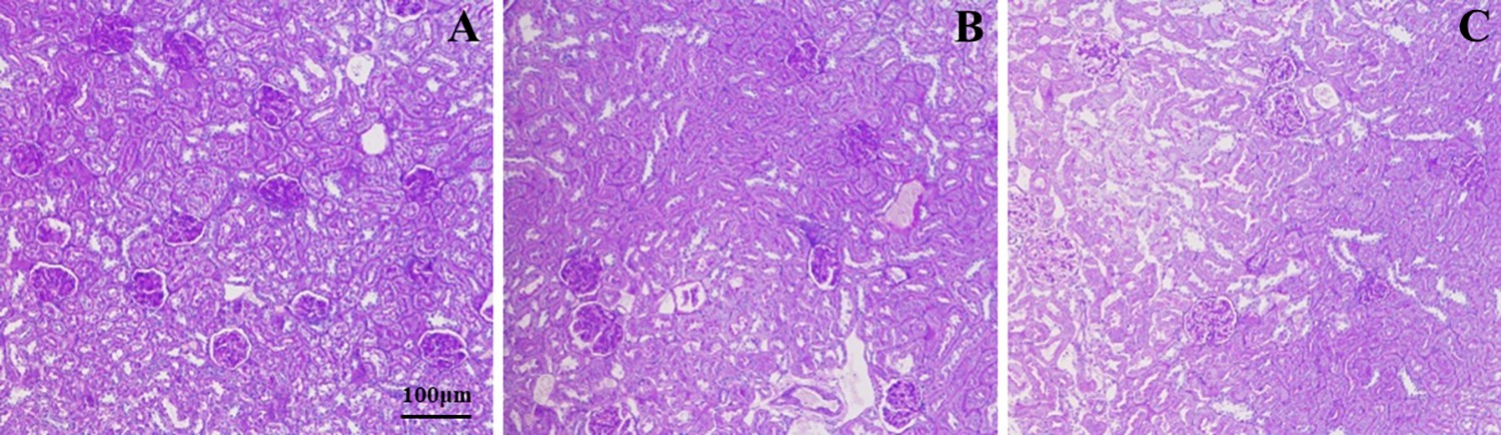
PAS staining of mesangial changes in the glomerulus. In both ovalbumin and gliadin groups, the glomerular mesangial was thickening, mesangial cell and matrix proliferation, and focal glomerulonephritis. A, Ovalbumin group; B, Gliadin group; C, Control group.

Immunofluorescence results demonstrated positive IgA and C_3_ staining in both ovalbumin and gliadin groups. Both model groups exhibited significant deposition of IgA and C_3_ in the skin, lung, brain, gastrointestinal tract, and the mesangial region of the glomerulus. Conversely, the control group yielded negative results (Figs 2 and 3). HE staining results indicated that the pathological changes observed in both ovalbumin and gliadin model groups were similar. In the renal tissue of the model groups, there was a significant presence of renal tubular epithelial cells exhibiting watery degeneration and loose cytoplasm, as well as eosinophilic flocs in the lumen. Additionally, congestion was observed in both the interstitium and glomerulus capillary (Fig 4A and 4B). In the gastric tissue of model groups, Focal necrosis and necrosis of gastric gland in the affected area were absent. However, there was evidence of inflammatory cell infiltration and eosinophil infiltration at the bottom of lamina proper. Additionally, there was smooth muscle necrosis in mucosal muscle and edema in submucosal tissue. The loose connective tissue exhibited a specific arrangement (Fig 4D and 4E). Intestinal tissue from the model groups exhibited extensive necrosis and shedding of intestinal villous epithelial cells into the mucosal layer, resulting in exposure of the lamina propria. Additionally, capillary extravasation was observed within the lamina propria, accompanying by infiltration of inflammatory cells (Fig 4G and 4H). In contrast, the control group displayed normal tissue structure (Fig 4C, 4F, and 4I). PAS staining of the kidney revealed thickening of the glomerular mesangium, with proliferation of both mesangial cell and matrix. Additionally, focal glomerulonephritis was observed in both ovalbumin and gliadin groups when compared to the control group (Fig 5).

### 3.3. Circulating cf-DNA levels and MPO-DNA in IgAV model

**Fig 6:**
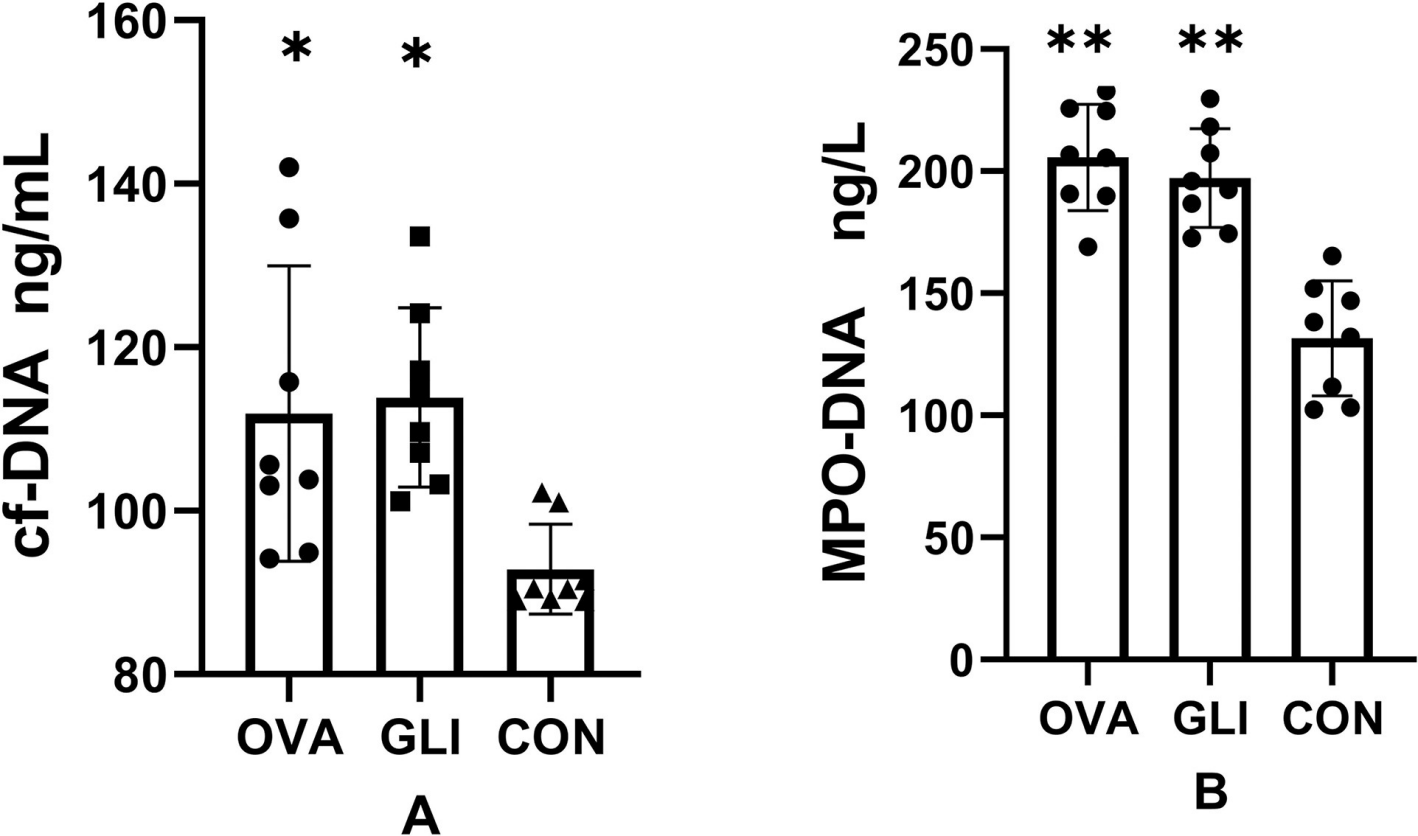
Circulating cf-DNA, MPO-DNA levels in IgAV models groups and control groups. cf-DNA (A), MPO-DNA (B) were elevated in the IgAV model groups compared to the control group (*P < 0.05, **P < 0.01). There were no differences between the gliadin group and ovalbumin group (P > 0.05). OVA, Ovalbumin group; GLI, Gliadin group; CON, Control group.

It is well-established that NETs consist of cf-DNA, MPO, NE, and histones, including citrullinated histone H3 (cit-H3). NETs are also the major source of circulating cf-DNA. To identify NETs, we quantified the levels of cf-DNA and MPO-DNA in plasma samples. The cf-DNA levels were 115.65 ± 19.62 ng/mL in the ovalbumin group, 111.35 ± 7.49 ng/mL in the gliadin group, and 92.86 ±5.48 ng/mL in the control group, with both model groups showing elevated circulating cf-DNA levels in compared to the control group (*P* < 0.05). However, there was no significant difference between the ovalbumin group and gliadin group as determined by post-hoc multiple comparison tests (Fig 6A). The MPO-DNA levels were 205.67 ± 21.8 ng/L in the ovalbumin group, 197.18 ± 20.22 ng/L in the gliadin group, and 131.51 ± 23.09 ng/L in the control group. The levels of MPO-DNA were elevated in both model groups compared to the control group (*P* < 0.05). There was no significant difference between the ovalbumin group and gliadin group (*P* > 0.05) (Fig 6B).

### 3.4. The fluorescence immunofluorescence results of NETs

**Fig 7:**
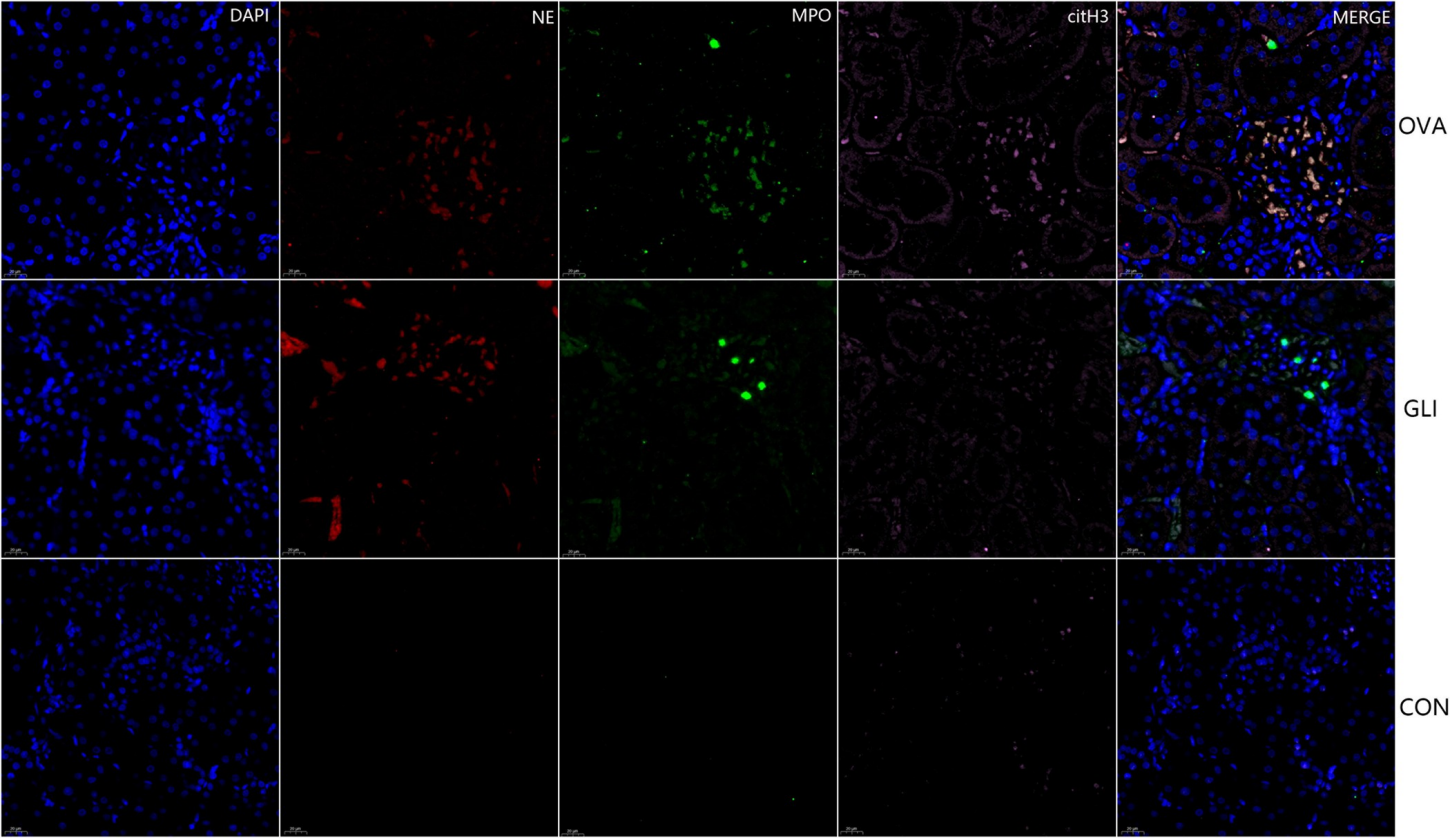
Multiple fluorescence staining of NETs in renal tissue. DAPI, blue; citH3, pink; NE, red; D, MPO, green. OVA, Ovalbumin group; GLI, Gliadin group; CON, Control group. Immunofluorescence staining for cit-H3, NE and MPO. DAPI was used for DNA staining and is shown in blue. NETs were labeled for extracellular DNA, and cit-H3 was seen to co-localize with MPO/NE in the mesangial area of the glomeruli.

**Fig 8:**
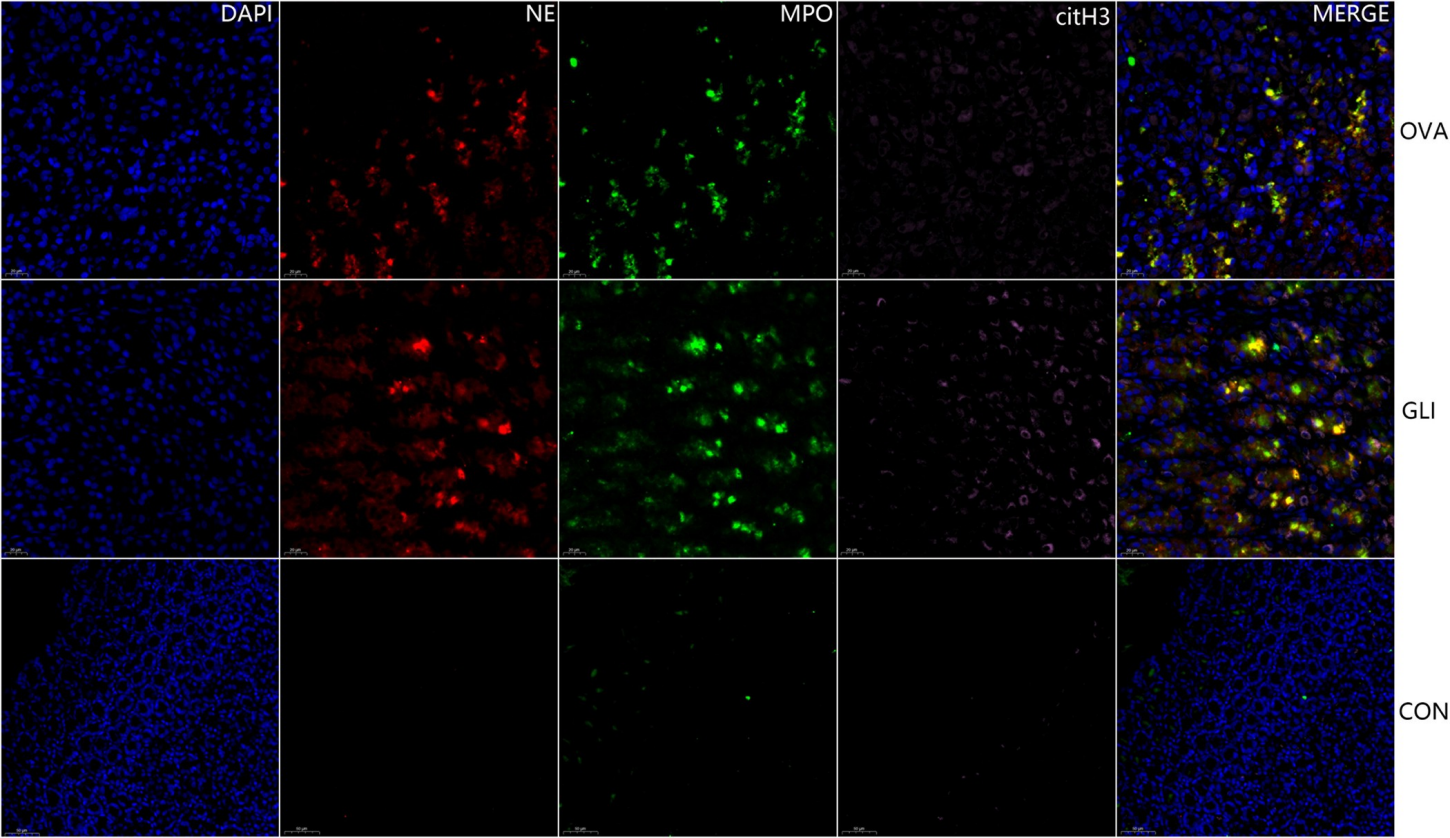
Multiple fluorescence staining of NETs in gastric antrum. DAPI blue; citH3, pink; NE, red; MPO, green. OVA, Ovalbumin group; GLI, Gliadin group; CON, Control group. Scale bars 20μm. NETs were present in the mucosa of gastric antrum of both IgAV model groups but absent in control group. Scale bars 20μm.

**Fig 9:**
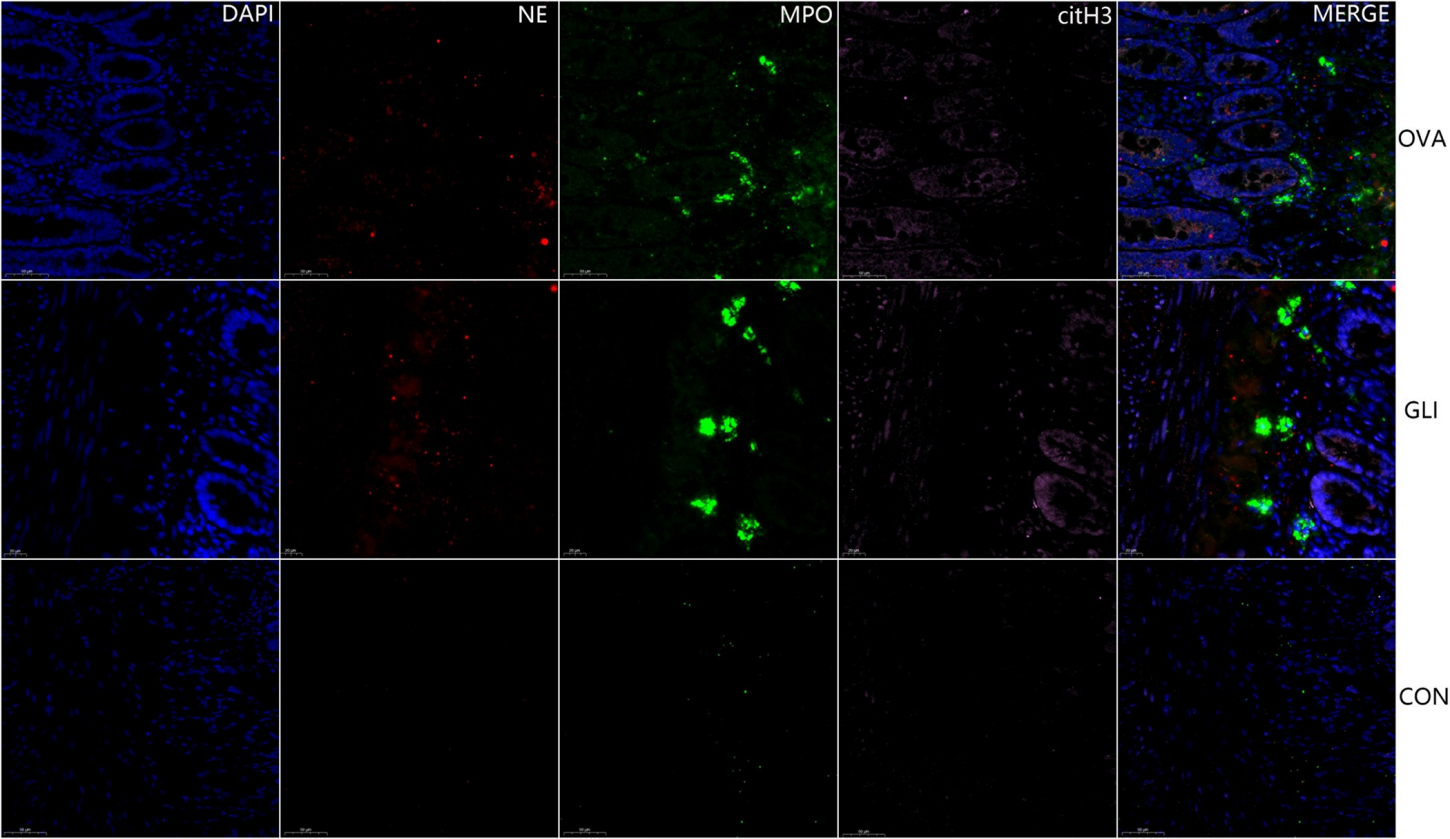
Multiple fluorescence staining of NETs in intestinal tissue. DAPI blue; citH3, pink; NE, red; MPO, green. OVA, Ovalbumin group; GLI, Gliadin group; CON, Control group. NETs were present in the villi mucosal and submucosal tissues of the duodenum of both IgAV model groups but absent in control group. Scale bars 20μm.

The results indicated the presence of NETs in both ovalbumin group and gliadin group, specifically within the affected gastric and duodenal mucosa tissues as well as mesangial regions of glomeruli. Immunofluorescence staining for cit-H3 (pink), NE (red) and MPO (green) confirmed the localization of NETs in these tissues. Furthermore, NETs were labeled for extracellular DNA, and cit-H3 was seen to co-localize with MPO/NE in the mesangial area of the glomeruli in both ovalbumin model group and gliadin model group (Fig 7). NETs were also present in the mucosa submucosal tissues of gastric antrum and duodenum in both IgAV model groups (Figs 8 and 9). There was no evidence of NETs formation or deposition in control group, which had normal renal and gastrointestinal histology (Figs 7–9). The typical characteristics of NETs in rats’ model were similar to those observed in IgAV patients [14].

## 4. Discussion

In this study, the establishment of IgAV rat models was compared using ovalbumin or gliadin combined with Indian ink and Freund’s complete adjuvant. The results demonstrated that deposition of IgA and C_3_ were observed in kidney, digestive tract, lung, and skin. Which is consistent with the pathology of IgAV patients [2, 17, 18]. In hematology analysis, both model groups exhibited elevated levels of the WBC, neutrophils, and platelet compared to the control group. These hematological changes are also commonly observed in most children with IgAV [19]. The hematological results between the two model groups were similar. The study showed that IgA was significantly increased while C3 was significantly reduced in both ovalbumin and gliadin groups, which is a characteristic feature of IgAV. All results were consistent with the previous study [16].

In a previous study, traditional Chinese medicine was administered via gavage, followed by OVA and Freund’s complete adjuvant to established IgAV rat and rabbit’s models [16]. Another IgAV animal model was established by using Indian ink and gliadin for the views that IgA1 is indued by gliadin as an important antibody of IgAV [20]. In this study, India ink was utilized for sealing the reticuloendothelial system, which distinguished it from previous study [9]. Furthermore, direct injection of OVA into the skin to induce local immune allergic reactions was avoided in this study due to some scholars believed that it cannot demonstrate systemic inflammatory responses in the skin. Besides, we have observed that OVA exhibits higher solubility and greater convenience for injection, while GLI presents significant challenges with respect to dissolution and time-consuming operation. Previously, it was believed that rats do not express Fc α receptor (FcαR) and therefore cannot established an IgAV model. However, recent studies have confirmed the expresses the Fc receptor regulated by the Fcamr gene in rats [4, 21, 22], providing a theoretical basis for studying the IgAV rat model.

In the current study, Indian ink was utilized to seal the reticuloendothelial system while gliadin or ovalbumin served as immune antigen for constructing the IgAV model. Gliadin and ovalbumin were originally employed in creating IgA nephropathy (IgAN) model [23–25]. However, the relationship between IgAV and IgAN remains incompletely understood despite their shared characteristics as multi-etiological related diseases influenced by genetic and environmental factors, IgA glycosylation abnormalities, as well as immune disorders [5, 24, 26]. The renal pathology of IgAV and IgAN is also similar [14]. Abnormal glycosylation of IgA and deposition of IgA are characteristic changes in both IgAN and IgAV. Some scholars believe that IgAV represents the systemic manifestation of IgAN in the course of disease progression [27]. Studies have also shown that leukocytoclastic vasculitis is found in IgAN [28–30]. Additionally, a study revealed elevated levels of IgA and IgA1 in children with IgAV, which were found to be correlated with gliadin as antigen [20]. Endothelial system clearance dysfunction is implicated in the pathogenesis of IgAV, which leads to deposition of immune complexes. Therefore, IgAV animal models using Indian inks and gliadin were reported. The second method employed in this study for constructing IgAV model involved the use of Indian ink, ovalbumin, and Freund’s complete adjuvant. Following sealing the reticuloendothelial system with Indian ink, Freund’s complete adjuvant was combined with ovalbumin to enhance the immune response. Ovalbumin was initially employed as an oral antigen to establish IgA-associated nephritis [25]. Subsequently, ovalbumin combined with Freund’s complete adjuvant to construct IgAV rat and rabbit model more efficiently [16].

Our study demonstrated the successful construction of IgAV rat models using both ovalbumin and gliadin. Notably, our administration of gliadin via intraperitoneal and intravenous routes differed from previous studies utilizing intragastric delivery. However, due to its poor solubility and time-consuming preparation, gliadin may present certain challenges in experimental design. Conversely, ovalbumin’s superior solubility renders it a more convenient option for researchers seeking to establish such models. Additionally, this study represents the first confirmation of NETs in IgAV rat model, providing a valuable tool for investigating the pathogenesis of NETs in IgAV, particularly IgAVN. The study demonstrated significant increases in cf-DNA and MPO-DNA, key components of NETs. Multiple fluorescent immunohistochemistry revealed the presence of NETs in kidney, stomach, and duodenal tissues while remaining negative in the control group. The results confirmed the potential involvement of NETs in the pathogenesis of IgAV rats. NETs have been confirmed to play a significant role in the pathogenesis of autoimmune diseases, including but not limited to SLE, psoriasis, arthritis, and small vasculitis [31–35]. The level of NETs was found to be elevated in patients with SLE, and a negative correlation was observed between the levels of DNase‐1 and NETs, suggesting that DNase‐1 may play a role in NET degradation [36]. Our previous study also confirmed the presence of NETs in IgAV patients, and we found a positive correlation between the activity of DNase I and the capacity for NET degradation [14]. The abnormal degradation of NETs attributed to the insufficient DNase I activity, leading to tissue damage. Another study has also confirmed the involvement of NETs in the pathogenesis of IgAV [15]. Insufficient clearance of NETs has been linked to the generation of autoantibodies in SLE, rheumatoid arthritis, and microvasculitis [10]. In ANCA-associated vasculitis, the presence of proteinase 3, MPO and lysosome‐associated membrane protein 2 antibodies can stimulate neutrophils to release NETs, exacerbating the injury caused by vasculitis [34, 37, 38]. Following the activation of anti-neutrophil cytoplasmic antibodies, neutrophils can induce the production of NETs. The components of NETs such as MPO, antigen proteases, possess specific target antigens that are presented to dendritic cells in order to stimulate a new immune response process. this subsequently leads to the formation of a vicious circle [17–19]. NETs can interact with inflammosomes and form pathological feedback loops, inducing neutrophils to release a large number of inflammatory mediators and factors [20–22]. The levels of NETs were found to be correlated with the severity of skin vasculitis in IgAV patients [13]. Our study showed that NETs are play a roly in the pathogenesis of IgAV. However, the precise mechanism by which NETs induce tissue damage in IgAV remains unclear.

There are certain limitations to this study: firstly, Although IgAV models can be constructed by ovalbumin or gliadin, only 25–40% of the rats developed skin rashes. This is a flaw in all current IgAV animal models, as the pathogenesis of IgAV is still not completely clear and the construction of the model was based solely on IgA and C_3_ complement deposition. Further studies focusing on the improving IgAV animal models will be necessary in the future. Secondary, it is known that NETs are involved in other autoimmune diseases such as SLE and lupus nephritis. However, few studies have focused on the relationship between NETs and other glomerulopathies. Further research is necessary to determine whether the involvement of NETs in pathogenesis are specific of IgAV or common also to other glomerulopathies such as IgAN and nephrotic syndrome.

In conclusion, both gliadin and ovalbumin combined with Indian inks can successfully establish IgAV rat models. Ovalbumin is more soluble and convenient for intravenous administration. This study confirmed the presence of NETs in the IgAV rat model for the first time, providing evidence on the mechanism involved in the pathogenesis of IgAV.

## Supporting information

S1 File (RAR)
